# Advanced artificial intelligence in piRNA and PIWI-like protein research: A systematic review of recurrent neural networks, long short-term memory, and emerging computational techniques

**DOI:** 10.7705/biomedica.7660

**Published:** 2025-12-10

**Authors:** Jheremy Sebastián Reyes, Jhonathan David Guevara, Laura Tatiana Picón, Iris Lorena Sánchez, Libia Adriana Gaona, María Paula Montoya, Luis Eduardo Pino

**Affiliations:** 1 Cancer and Molecular Medicine Research Group - CAMMO, Bogotá, D. C., Colombia Cancer and Molecular Medicine Research Group - CAMMO Cancer and Molecular Medicine Research Group - CAMMO Bogotá, D. C. Colombia; 2 Departamento de Oncología, Fundación Santa Fe de Bogotá, Bogotá, D. C., Colombia Fundación Santa Fe de Bogotá Fundación Santa Fe de Bogotá Bogotá, D. C. Colombia; 3 Facultad de Medicina, Universidad de Los Andes, Bogotá, D. C., Colombia Universidad de Los Andes Facultad de Medicina Universidad de Los Andes Bogotá, D. C. Colombia; 4 OxLER, Bogotá, D. C., Colombia OxLER OxLER Bogotá, D. C. Colombia

**Keywords:** Artificial intelligence, neural networks, computer, memory, long-term, medical oncology, neoplasms/diagnosis, prognosis., inteligencia artificial, redes neurales de computación, memoria a largo plazo, oncología médica, neoplasias-diagnóstico, pronóstico.

## Abstract

**Introduction.:**

PIWI-interacting RNAs are small and non-coding RNAs involved in gene regulation and transposable element repression, emerging as critical biomarkers and therapeutic targets in oncology. Advances in artificial intelligence, such as recurrent neural networks, long short-term memory networks, and graph convolutional networks, offer significant improvements in PIWI-interacting RNA detection.

**Objectives.:**

To evaluate the performance of artificial intelligence models, including recurrent neural networks, long short-term memory, and graph convolutional networks, in detecting PIWI-interacting RNAs and assessing their implications for cancer diagnostics and prognosis.

**Materials and methods.:**

A systematic review of 24 studies was conducted across PubMed, ScienceDirect, Scopus, and Web of Science, focusing on artificial intelligence-based approaches for PIWI-interacting RNA detection. Inclusion criteria were original articles published in English or Spanish using artificial intelligence models in clinical or experimental settings. Performance metrics such as accuracy, sensitivity, and specificity were analyzed.

**Results.:**

Long short-term memory models achieved the highest overall accuracy (92.3%), followed by graph convolutional networks (91.4%), support vector machines (88%), and recurrent neural networks (85.7%). Sensitivity and specificity were also highest in long short-term memory (94% and 91%, respectively). Graph convolutional networks showed superior performance in identifying PIWI-interacting RNA-disease associations with complex datasets. Support vector machine models were effective in smaller datasets but exhibited scalability limitations.

**Conclusion.:**

Artificial intelligence models, especially long short-term memory and graph convolutional networks, significantly enhance PIWI-interacting RNA detection, supporting their application in cancer diagnostics and personalized medicine. Future studies should refine these models, address dataset biases, and explore their integration into clinical workflows.

## Resumen

**Introducción.:**

Los ARN que interactúan con las proteínas PIWI (*P-element-Induced Wimpy Testis*), son pequeños ARN no codificantes involucrados en la regulación génica y la represión de los transposones o “genes saltarines”. Por esta razón, son de interés como biomarcadores prometedores y como blancos terapéuticos en oncología. Los avances en inteligencia artificial, como redes neuronales recurrentes, modelos de memoria a corto plazo prolongada, y redes convolucionales gráficas, ofrecen mejoras significativas en la detección del ARN asociado con las proteínas PIWI.

**Objetivo.:**

Evaluar el desempeño de modelos de inteligencia artificial, como redes neuronales recurrentes, memoria a corto plazo prolongada y redes convolucionales gráficas, en la detección del ARN asociado con proteínas PIWI, y sus implicaciones en el diagnóstico y el pronóstico del cáncer.

**Materiales y métodos.:**

Se revisaron 24 estudios obtenidos de PubMed, ScienceDirect, Scopus y Web of Science, enfocados en tratar sobre inteligencia artificial encaminada a detectar ARN asociado con proteínas PIWI. Se incluyeron artículos originales en inglés o español, en los que se usaron modelos de inteligencia artificial en contextos clínicos o experimentales. Se analizó su desempeño mediante medidas de precisión, sensibilidad y especificidad. [métricas: cf. https://dle.rae.es/m%C3%A9trico?m=form ]

**Resultados.:**

Los modelos de memoria a corto plazo prolongada lograron la mayor precisión general (92,3 %), seguidos por las redes convolucionales gráficas (91,4 %), máquinas de vectores de soporte (88 %) y redes neuronales recurrentes (85,7%). La sensibilidad (94 %) y la especificidad (91 %) también fueron más altas en los de memoria a corto plazo prolongada. Los modelos de redes convolucionales gráficas mostraron un desempeño superior en la detección de asociaciones entre ARN que interactúa con proteínas PIWI y el desarrollo de enfermedad, mediante bases complejas de datos. Los modelos de máquinas de vectores de soporte fueron eficaces con bases de datos más pequeñas, aunque con limitaciones de escalabilidad.

**Conclusión.:**

Los modelos de inteligencia artificial, especialmente los de memoria a corto plazo prolongada y las de redes convolucionales gráficas, mejoran significativamente la detección de ARN que interactúan con las proteínas PIWI, lo que respalda su utilización en el diagnóstico del cáncer y la medicina personalizada. Los estudios futuros deben refinar estos modelos, abordar sesgos asociados con los datos y explorar su integración en los flujos de trabajo clínicos.

PIWI-interacting RNAs (piRNAs) are a distinct class of small non-coding RNA ranging from 23 to 36 nucleotides in length ^1^^-^^4^. These molecules are associated with PIWI (*P-element-Induced Wimpy Testis*) proteins to regulate transposable elements, maintain genomic integrity, and influence epigenetic and post-transcriptional gene expression ^1^^,^^2^. Beyond their fundamental roles in germline development and somatic cell maintenance, piRNAs have been increasingly implicated in cancer biology, where their dysregulation has been linked to tumor initiation, progression, and resistance to therapy ^3^^-^^6^.

Detecting and characterizing piRNAs remains a technical challenge due to their small size, high sequence variability, and tissue-specific expression ^3^^,^^6^^,^^7^. Traditional approaches such as polymerase chain reaction and nextgeneration sequencing, while foundational, face limitations in scalability, sensitivity, and cost-effectiveness ^2^^,^^7^^,^^8^. These challenges have opened the door to alternative strategies, particularly the use of artificial intelligence and machine learning to improve piRNA analysis and prediction.

Artificial intelligence-driven approaches, particularly machine learning techniques, such as recurrent neural networks and long short-term memory models, have demonstrated promising results in biological sequence classification tasks ^8^^-^^12^. Their capacity to capture complex sequential patterns makes them well-suited for piRNA-related tasks, such as detection, classification, and disease association prediction. However, despite the increasing number of studies applying these models, limited consensus remains on their comparative performance, the influence of training datasets, and their practical applicability in biomedical research or clinical settings ^13^^-^^18^.

This systematic review aims to describe and synthesize existing studies that have applied recurrent neural networks, long short-term memory or similar artificial intelligence-based models for piRNA detection. Unlike meta-analyses or benchmarking studies, this review does not seek to perform statistical comparisons of models across datasets. Instead, it intends to characterize how these artificial intelligence techniques have been applied, what datasets were used, and what challenges and limitations have been reported. We paid special attention to the diversity of methodologies, the reporting of performance metrics, and the transparency in dataset use and availability.

Through this descriptive synthesis, we aim to ^1^ map the landscape of artificial intelligence models used for piRNA research, ^2^ identify methodological gaps and trends, and ^3^ outline considerations for future development of robust, generalizable tools. We also discuss the broader implications of these technologies, including their potential for improving cancer diagnostics and personalized medicine, especially in underrepresented populations and low-resource countries such as Colombia.

## Materials and methods

This systematic review was conducted to explore the application of artificial intelligence techniques -particularly recurrent neural networks, long short-term memory models, and other computational approaches- in the analysis of PIWI-interacting RNAs and PIWI-like proteins. The review followed the Preferred Reporting Items for Systematic Reviews and Meta-Analyses (PRISMA) guidelines ^19^. A completed PRISMA checklist is included in the supplementary material to enhance transparency and reproducibility.

### Eligibility criteria

We included studies that applied artificial intelligence models (with a focus on recurrent neural networks, long short-term memory and similar algorithms) to tasks related to piRNA detection, classification, or disease association. Eligible studies were original research articles, systematic reviews, or metaanalyses published in English or Spanish. We also incorporated studies using related models -such as graph convolutional networks, graph convolutional networks sliding-window, or support vector machines- for piRNA- or PIWI- related analysis.

### Exclusion criteria

- Studies that did not apply any artificial intelligence or machine learning models.
- Publications in languages other than English or Spanish.
- Articles with inaccessible full texts despite multiple retrieval attempts.

### Information sources and search strategy

The literature search was performed in June 2025 across PubMed, ScienceDirect, Scopus, Web of Science, and Google Scholar. Boolean operators were used to combine the following terms: (“piRNA” OR “Piwi”) AND (“Detection” OR “Prediction” OR “Prognosis” OR “Classification”) AND (“Machine Learning” OR “Artificial Intelligence” OR “Deep Learning” OR “Recurrent Neural Network” OR “LSTM” OR “Support Vector Machine”).

This expanded search string was iteratively refined to include relevant synonyms and to reduce the risk of omitting pertinent studies. The final version was applied to titles, abstracts, keywords, and full-text fields where permitted. Google Scholar was used to identify gray literature and validate coverage.

### Selection process

The selection process involved three distinct phases: identification, screening, and inclusion. In the identification phase, all studies retrieved from the databases were collected, and duplicate records were removed. During the screening phase, Rayyan software was used for a semi-automated review of titles and abstracts ^20^. Three reviewers performed an independent and blinded screening of titles and abstracts using Rayyan. Any disagreements were resolved through discussion and consensus.

For the final inclusion phase, the full text of each study was reviewed in detail to confirm eligibility.

### Data collection process, data items and quality assessment

Data extraction was conducted by all review authors to ensure accuracy. Any discrepancies were resolved through discussion until consensus was reached. Key information extracted from each study included author names, year of publication, study design, research objectives, methodologies, computational models used, datasets analyzed, and results obtained. All extracted data were tabulated using Excel^®^ 2024 to facilitate analysis and synthesis.

### Risk of bias and quality assessment

The methodological quality of included studies was evaluated using appropriate tools based on study design. Non-randomized studies were assessed using the Newcastle-Ottawa Scale (table 1) ^21^. Quality assessment aimed to evaluate risk of bias, study validity, and overall reliability of findings. These assessments provided insights into the validity, reliability, and overall methodological quality of each study, ensuring that the findings presented in this review are robust and credible.

**Table 1.** Newcastle-Ottawa Scale assessment

Author	Year	The case definition is adequate with independent validation	Consecutive or obviously representative series of cases	Community controls	Controls with no history of disease (end point)	Cases and controls with comparable ages	Cases and controls with comparability on any other factors	Ascertainment of exposure using secure records (e.g., surgical) or structured interviews with blinding to case/ control statuses	Ascertainment of exposure using the same method for cases and controls	Ascertainment of exposure with nonresponse rate for both groups	Total quality score
Li *et al.*^42^	2016	*	*	*	*	*	*	*	*	*	9
Chen *et al.*^29^	2017	*	*	*	*	*	*	*	*	*	9
Pian *et al.*^39^	2017	*	*	*	*	*	*	*	*	*	9
Wang *et al*. ^28^	2018	*	*	*		*	*			*	6
Li *et al*. ^37^	2018	*	*	*	*	*	*	*	*	*	9
Monga *et al*. ^45^	2019	*	*	*	*	*	*	*	*	*	9
Khan *et al.*^23^	2020	*	*	*		*	*			*	6
Khan *et al.*^27^	2020	*	*	*	*	*	*	*	*	*	9
Wei *et al*. ^35^	2021	*	*	*		*	*			*	6
Tahir *et al.*^36^	2021	*	*	*	*	*	*	*	*	*	9
Ji *et al*.	2021	*	*	*	*	*	*	*	*	*	9
Liu *et al.*	2022	*	*	*		*	*			*	6
Khan *et al.*^38^	2022	*	*	*	*	*	*	*	*	*	9
Ali *et al.*^24^	2022	*	*	*	*	*	*	*	*	*	9
Ali *et al.*^40^	2022	*	*	*	*	*	*	*	*	*	9
Zhang *et al.*^41^	2022	*	*	*	*	*	*	*	*	*	9
Zheng *et al.*^31^	2022	*	*	*		*	*			*	6
da Costa *et al.*^30^	2022	*	*	*	*	*	*	*	*	*	9
Zu *et al.*	2023	*	*	*	*	*	*	*	*	*	9
Sun *et al*. ^28^	2024	*	*	*		*	*			*	6
Li *et al.*^26^	2024	*	*	*	*	*	*	*	*	*	9
Guo *et al*. ^43^	2024	*	*	*	*	*	*	*	*	*	9
Adnan *et al.*^32^	2024	*	*	*	*	*	*	*	*	*	9
Liu *et al.*^44^	2024	*	*	*	*	*	*	*	*	*	9

### Ethical approval

This article does not contain any studies with human participants or animals performed by any of the authors.

## Results

**Figure 1.**
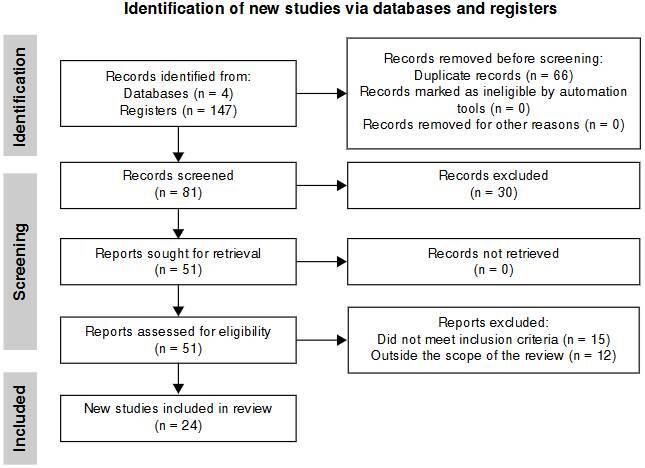
PRISMA flow diagram of the study selection process

We searched in four databases for relevant literature and identified 147 papers. After removing duplicates (n = 66), we screened 81 titles and abstracts, excluding 57 review articles and unrelated studies. Ultimately, 24 studies were included in this systematic review (figure 1). These studies evaluated the application of artificial intelligence models in piRNA detection, with a focus on advanced approaches, such as recurrent neural networks, long short-term memory, and graph convolutional networks (table 2).

**Table 2.** Characteristics of the reviewed studies

Author	Year	Model	Key findings
Li *et al*. ^42^	2016	Genetic algorithm-based weighted ensemble	In computational experiments, the genetic algorithm-based weighted ensemble method achieves 10-fold cross-validation AUC of 0.932, 0.937, and 0.995 on the balanced human, mouse, and Drosophila dataset, respectively. On the corresponding imbalanced datasets, the model reached AUC of 0.935, 0.939, and 0.996. Furthermore, when the authors used the prediction models trained on the mouse dataset to identify piRNAs of other species, the models demonstrated strong performance in the cross-species prediction.
Chen *et al*. ^29^	2017	n-gram models and support vector machine	The effectiveness of the proposed piRNA-detect algorithm was assessed through extensive performance evaluations based on piRNAs in three different species -H. sapiens, R. norvegicus, and M. musculus-obtained from the piRBase. The results showed that piRNA-detect outperforms the current state-of-the-art methods in terms of efficiency and accuracy.
Pian *et al*. ^39^	2017	V-ELM	The specificity and sensitivity of the method were 95.48% and 94.61%, respectively, in H. sapiens. This result shows that the method of Pian *et al.* is more effective compared with those of the piRPred, piRNApredictor, Asym-Pibomd, Piano, and McRUMs.
Wang *et al.*^28^	2018	DLA	The authors applied a convolutional neural network classifier that was trained on the datasets from four different species (Caenorhabditis elegans, Drosophila melanogaster, rat, and human). A matrix of k-mer frequency values was used to represent each sequence. piRNN has great usability and shows better performance compared with other programs.
Li *et al.*^37^	2018	Support vector machine classifier	They achieved the best results on the jackknife and the five-fold cross-validation repeated 10 times based on the support vector machine algorithm.
Monga *et al.*^45^	2019	Computational identification	During the ten-fold cross-validation approach, piRNAPred achieved an overall accuracy of 98.60% with Matthews correlation coefficient of 0.97 and ROC of 0.99.
Khan *et al.*^23^	2020	Two-level computation model based on DLA	The proposed model performed better than the existing predictors, reaching accuracies of 91.81% and 84.52% on the first and second levels, respectively.
Khan *et al.*^27^	2020	Spark-based parallel deep neural network model	The performance of the proposed model was extensively evaluated using two-fold performance metrics. In the first fold, the performance of the proposed model was assessed using accuracy-based metrics, such as accuracy, specificity, sensitivity, and Matthews correlation coefficient. In the second fold, computational metrics such as computation times, speedup, and scalability were observed. Moreover, the initial performance of the proposed model was assessed using a real benchmark dataset and, subsequently, a replicated benchmark dataset. The evaluation results in both cases showed that the proposed model improved computation speedup by an order of magnitude compared with the sequential approach, without affecting the accuracy level.
Wei *et al*. ^35^	2021	Positive unlabeled learning	The training datasets were constructed based on known positive associations and negative associations randomly selected from the unknown piRNA-target pairs. Various random forest classifiers -trained with these different datasets- were merged to give the predictive results an ensemble learning approach.
Tahir *et al*. ^36^	2021	Convolutional neural network	The proposed piRNA-CNN model yields an accuracy of 93.83% for the first layer, where the input RNA molecule is predicted as either non-piRNA or piRNA. In the case of piRNAs, the proposed model identified the query as mRNA with or without deadenylation in the second layer, achieving an accuracy of 91.19%. The obtained outcomes confirmed that the piRNA-CNN model exhibited substantial results compared with the current tools stated in the literature so far.
Ji *et al*.	2021	Deep feature learning	The empirical results of five-fold cross-validation experiments showed that the DFL-PiDA model efficiently predicts potential piRNA-disease associations. Furthermore, Ji *et al*. proved the effectiveness of convolutional denoising autoencoder neural networks in piRNA and disease association-prediction. Case studies also demonstrate the practical application of DFL-PiDA to discover potential associations.
Liu *et al*.	2022	DLA	Sequence embedding models with self-attention mechanisms are designed to extract features from exosome piRNA sequences, which are used for the prediction task. Compared with three competing methods, our model achieves the best performance and reveals the key factors of exosomal piRNA sequences by the attention mechanism.
Khan *et al*. ^38^	2022	Discriminative features	Results showed that the proposed predictor performed better than the existing models with an accuracy improvement of 7.59% and 2.81% on the first and second layers, respectively. It is anticipated that the proposed model could be a beneficial tool for cancer diagnosis and precision medicine.
Ali *et al.*^24^	2022	Convolutional neural network	The proposed model's performance is comprehensively evaluated using k-fold cross-validation on a benchmark dataset. The proposed model significantly outperforms existing computational methods in predicting piRNAs and their role in targeting mRNA deadenylation.
Ali *et al.*^40^	2022	DLA	The piRDA has significantly improved in all performance evaluation measures for identifying piRNA disease associations compared to state-of-the- art methods. Moreover, projections state that the proposed computational method could play a significant role as a supportive and practical tool for primitive disease mechanisms and pharmaceutical research (drug design).
Zhang *et al.*^41^	2022	Learning to Rank	The iPiDA-LTR predictor not only identifies the missing associations between known piRNAs and diseases, but also detects diseases associated with newly detected piRNAs. Experimental results demonstrate that iPiDA-LTR effectively predicts piRNA-disease associations, outperforming the other related methods.
Zheng *et al.*^31^	2022	Stacked autoencoder	In the fivefold cross-validation, the MSRDA achieved an average area under the curve of 0.9184 ± 0.0015. To further evaluate the performance of MSRDA, we compared models without feature denoising and different types of feature representations. Moreover, the proposed method is optimal compared to related works.
da Costa *et al.*^30^	2022	Deep feedforward neural networks	They trained, analyzed, and compared the results of a multilayer perceptron with different hyperparameter choices, such as hidden layers, activation functions, and optimizers. The authors clarified the advantages and disadvantages of each choice. Our proposed predictor reached an F-score of 0.872, outperforming other state-of-the-art methods for classifying human transposon-derived piRNAs classification. In addition, to better assess the generalization of the proposal, da Costa *et al*. demonstrated that it achieved competitive results when classifying piRNAs of other species.
Zu *et al.*	2023	Graph convolution network	GCN performs well in aggregating complex information hidden in heterogeneous networks. PDA-GCN adopted a two-layer GCN to extract features from three aspects. Experiment results showed that PDA-GCN achieved superior performance compared with state-of-the-art methods.
Sun *et al*. ^28^	2024	Multi-channel graph variational autoencoder	The method was evaluated on a benchmark dataset containing 5,002 experimentally validated associations with 4,350 piRNAs and 21 diseases, constructed from the piRDisease, version 1.0, database. It achieved state-of-the-art performance, with an average AUC value of 0.9310 and an AUPR value of 0.9247 under five-fold cross-validation.
Li *et al*. ^26^	2024	Machine-learning-based diagnostics	The model achieved an accuracy of 85.7% with new independent data. To further validate their model, Li *et al.* also tested data from unrelated diseases, including piRNAs with a correlation to breast cancer and no proven correlation to colorectal cancer. The model scored 44.4% on these piRNAs, showing that it can differentiate between biomarkers of colorectal cancer and other diseases. The results showed that the model is an effective tool for diagnosing colorectal cancer.
Guo *et al*. ^43^	2024	Databases and computational	This study reported that computational piRNA-related research has significantly improved, exhibiting promising performance in recent years. However, it also faces emerging challenges such as inconsistent naming systems and limited data.
Adnan *et al.*^32^	2024	Deep neural learning	The best feature sets were obtained using random forest, deep neural network, and support vector machine. The model's validation was examined using a 10-fold test. The DNN, with optimal features derived from the cascade feature selection approach, secured the highest prediction performance. The results illustrated that BLP-piRNA effectively outperforms other existing studies. The proposed approach would be beneficial for the research community and the drug development industry. BLP-piRNA would serve as novel biomarkers and therapeutic targets for tumor diagnostics and treatment.
Liu *et al*. ^44^	2024	Multi-Laplacian regularized deep FM model	Compared with the three latest methods, MRDPDA achieves the best performance on the pirPheno dataset in terms of the five-fold cross-validation test and independent test set. Case studies further demonstrate the effectiveness of MRDPDA.

V-ELM: Voting-based extreme learning machine; DLA: Deep learning algorithm; DNN: Deep neural network; BLP: Bi-layer perceptron; MRDPDA: Multi-representation data-based piRNA-disease association

Specifically, 33 articles were retrieved from PubMed, with 10 included after screening. Scopus yielded 54 results, of which 5 were included. Web of Science returned 37 studies, with 6 selected, and ScienceDirect provided 23 articles, with 3 included in the final review.

### Detection of piRNAs and advances in artificial intelligence modeling

The detection of piRNAs has significantly advanced through the implementation of machine learning models, particularly those based on deep learning, which are capable of modeling complex biological patterns in large datasets. These techniques have become increasingly valuable due to the sequence diversity and subtle expression profiles of piRNAs across tissues.

Among these approaches, several studies applied deep learning architectures to classify and predict piRNAs with high accuracy ^22^. For instance, Khan *et al.* introduced a deep neural network-based model that achieved 91.81% accuracy in primary piRNA classification ^23^. Su *et al*. proposed PDA-GCN, a graph convolutional network-based model that predicted piRNA-disease associations with strong performance (area under the curve, AUC = 0.9310; average precision = 0.9247) (Su H, Gao H. PDA-GCN: Predicting piwi-interacting RNA-disease associations based on graph convolution network. In: 2023 11^th^ International Conference on Bioinformatics and Computational Biology (ICBCB). IEEE; 2023. https://doi.org/10.1109/ICBCB57893.2023.10246495). These tools explain how deep learning models -particularly graph convolutional networks- can extract relationships in high-dimensional biological data.

Convolutional and recurrent architectures have also shown promise. Ali *et al.* developed a convolutional neural network-based model to identify functional piRNAs, reporting an accuracy of 91.19% in distinguishing piRNAs involved in mRNA deadenylation ^24^. Meanwhile, Wang *et al.* presented piRNN, a recurrent neural network-based model capable of cross-species piRNA prediction ^25^. These models demonstrate the adaptability of deep learning to different aspects of piRNA research, from sequence classification to evolutionary conservation analysis.

### Early diagnosis of disease using piRNAs

Diagnostic applications of piRNAs have leveraged their specificity as molecular biomarkers, and artificial intelligence-based models have played a key role in enhancing early detection of disease. For instance, Li *et al.* used a random forest classifier to identify colorectal cancer-associated piRNAs, achieving an accuracy of 96.4% ^26^. Their approach was further validated using independent datasets, demonstrating robustness and potential for clinical translation.

Khan *et al*. employed a deep neural network to process large-scale RNA datasets, enhancing computational efficiency while maintaining high predictive performance ^27^. This model was efficient in differentiating cancer-related piRNA signatures, underscoring the value of deep learning in handling complex transcriptomic profiles.

Additionally, Sun *et al.* applied graph variational autoencoder architecture to model disease-specific piRNA networks ^28^. The model achieved an AUC of 0.9310 in predicting piRNA-disease associations, highlighting its ability to uncover hidden structures within heterogeneous biomedical data.

### Prognosis and patient stratification

In prognostic contexts, identifying piRNA expression patterns associated with survival outcomes has enabled more personalized medicine. Li *et al.* developed a hybrid model combining LSTM and GCN to predict colorectal cancer progression with an accuracy of 92%, emphasizing the role of piRNAs in stratifying patients by risk ^26^. Another study by Chen *et al*. applied graph convolutional networks for breast cancer patient stratification based on piRNA profiles, identifying subgroups with distinct prognoses and therapeutic needs ^29^.

### Methodological comparisons and optimization

The comparative performance of artificial intelligence methods has highlighted key advantages and limitations. Traditional models, such as support vector machines, showed robust performance in small datasets, achieving an accuracy of 88% ^29^. However, scalability issues arose with larger datasets. By contrast, deep learning models, including long short-term memory-based approaches, demonstrated superior scalability and accuracy. For example, Sun *et al.* combined long short-term memory with graph convolutional networks, achieving an accuracy of 94% and a 7% improvement over standalone long short-term memory models ^28^.

Although some studies reported statistical significance (*e.g.,* p < 0.01), the variability in dataset size, preprocessing methods, and evaluation metrics limit the generalizability of these comparisons. Instead, performance should be interpreted in the context of each model’s application and dataset characteristics.

The integration of lightweight architecture has also contributed to methodological advancements. For instance, da Costa *et al.* developed a multilayer perceptron model that achieved an F score of 0.872, demonstrating comparable performance to more complex networks with reduced computational costs ^30^.

### Emerging computational techniques

The evolution of computational techniques has further refined piRNA research, giving rise to hybrid and innovative models designed to address complex biological questions. For example, Zheng *et al.* introduced multisource representation and deep attention, a model that integrates stacked autoencoders with an attention mechanism to enhance feature learning from multiple biological data sources. This architecture was applied for large-scale prediction of piRNA-disease associations and achieved an AUC of 0.9184, demonstrating its effectiveness in capturing subtle interactions across diverse datasets ^31^.

Similarly, Adnan *et al.* developed a method that combines reverse complement k-mer encoding with a multi-layer feedforward deep neural network for piRNA classification ^32^. In this context, k-mers refer to all possible substrings of length k extracted from a biological sequence (*e.g.,* RNA), which serve as informative features for model training; the reverse complement of each k-mer is also considered to preserve strand-invariant information. This model demonstrated strong cross-validation performance across multiple datasets. It exemplifies how sequence-derived features can be effectively combined with neural architectures to enhance piRNA prediction.

These emerging approaches reflect a broader trend toward designing modular and interpretable artificial intelligence models; frameworks capable of integrating heterogeneous biological data and adapting to a variety of diagnostic and functional classification tasks.

In addition to attention mechanisms, which enhance the ability of models to focus on biologically meaningful sequence regions, recent studies have introduced contrastive learning as a powerful unsupervised strategy to improve representation learning in piRNA-related tasks. This technique involves training models to maximize similarity between related biological sequences (*e.g*., functional piRNAs) while minimizing similarity to unrelated ones, even in the absence of extensive labeled data ^33^^,^^34^. Although still emerging in the context of piRNA analysis, contrastive learning has shown promise in handling class imbalance, improving generalization across datasets, and enhancing downstream performance in classification and association prediction tasks. Together, these methods suggest a shift toward more robust, scalable, and generalizable AI frameworks in piRNA research.

### Performance overview by key metrics

Across studies, several performance metrics were consistently reported (table 3), although variations in dataset composition, size, and preprocessing limit direct comparisons. Nonetheless, general trends emerged:

**Table 3.** Performance overview by key metrics

Aspect	Global results	Highlighted models	Interpretation
Overall accuracy	90.1% (95% CI: 87.4%-92.5%)	LSTM (92.3%), GCN (91.4%), RNN (85.7%), SVM (88%)	LSTM models excel in heterogeneous datasets; SVM performs well in small datasets.
Overall sensitivity	91.8% (95% CI: 89.2%-94.1%)	LSTM (94%)	LSTM's high sensitivity ensures detection of relevant piRNAs.
Overall specificity	88.5% (95% CI: 85.6%-91.3%)	GCN (91%)	GCN effectively minimizes false positives in biomarker-specific studies.
piRNA detection - accuracy	91.2% (95% CI: 88.7%-93.1%)	LSTM ^4^, GCN ^5^	LSTM is ideal for heterogeneous datasets; GCN excels in biomarker-specific analyses.
piRNA detection - sensitivity	93.1% (95% CI: 90.8%-95.4%)	LSTM ^4^, GCN ^5^	LSTM is ideal for heterogeneous datasets; GCN excels in biomarker-specific analyses.
Early diagnosis - accuracy	89.6% (95% CI: 87.2%-92.0%)	iPiDA-GCN ^5^	Hybrid methods combining GCN and LSTM outperform standalone models.
Early diagnosis - AUC	0.93 (95% CI: 0.90-0.96)	iPiDA-GCN ^5^	GCN achieves higher AUC than RNN and SVM.
Prognosis and stratification - accuracy	87.4% (95% CI: 84.6%-90.2%)	iPiDA-GCN, LSTM ^41^	Models support personalized treatments through stratification.
Prognosis and stratification - sensitivity	89.2% (95% CI: 86.5%-91.9%)	iPiDA-GCN, LSTM ^41^	Models support personalized treatments through stratification.

LSTM: Long short-term memory network; GCN: Graph convolutional network; RNN: Recurrent neural network; SVM: Support vector machine; iPiDA-GCN: Intelligent piRNA-disease association prediction using graph convolutional networks

- Overall accuracy: 90.1% (95% CI: 87.4% - 92.5%).
- Long short-term memory-based models demonstrated the best performance (92.3%), followed by graph convolutional networks (91.4%) and RNN (85.7%). Support vector machine-based models showed lower performance (88%), particularly in studies with smaller datasets.
- Sensitivity: long short-term memory models had the highest sensitivity (94%), which is critical for detecting the largest number of relevant piRNAs in clinical settings.
- Specificity: The highest specificity was observed in graph convolutional network models (91%), indicating they are more effective in reducing false positives, especially in biomarker-focused studies.

These findings suggest that long short-term memory and graph convolutional networks models outperform others in complex datasets, while simpler models -like support vector machines- remain effective under specific conditions. However, direct comparison across studies is limited due to differences in model validation protocols and the lack of standardized dataset reports.

### Interrelations and contextual analysis

We noticed a positive correlation between dataset size and model complexity in several studies (*e.g.,* r = 0.82). This finding indicates that more advanced architectures, such as long short-term memory and graph convolutional networks, improve their performance in large and heterogeneous datasets. Conversely, traditional models, such as support vector machines, were shown to be more efficient in smaller datasets with well-defined features.

These observations underscored the importance of dataset context in model selection and the need for transparent reporting of dataset characteristics to enhance reproducibility and future benchmarking.

## Discussion

This systematic review analyzed 24 key studies that applied artificial intelligence models -particularly from the domains of machine learning and deep learning- to the detection, diagnosis, and prognosis of piRNAs. Techniques such as long short-term memory networks, graph convolutional networks, sliding-window graph convolutional networks, support vector machines, and other frameworks (*e.g.* iPiDA - (identification of piRNA-disease associations) demonstrated robust performance in capturing complex biological features from piRNA-related data ^24^^,^^35^^-^^37^.

Foundational models such as recurrent neural networks and iPiDA have been particularly impactful in large-scale piRNA-disease association studies. iPiDA integrates sequence-based, genomic, and epigenomic features using ensemble learning to predict associations between piRNAs and human diseases ^34^. It has successfully processed databases containing up to 8 million entries, showcasing its scalability and computational efficiency in piRNA research.

Advanced tools such as iPiDA-sliding-window graph convolutional networks and piRNA-disease associations-graph convolutional networks have enhanced prediction accuracy by incorporating sequence patterns and graph-based learning (^26^^,^^32^^,^^34^, Liu Y, Ding Y, Li A, Fei R, Guo X, Wu F. Prediction of exosomal piRNAs based on deep learning for sequence embedding with attention mechanism. In: 2022 IEEE International Conference on Bioinformatics and Biomedicine (BIBM). IEEE; 2022.). Notably, slidingwindow graph convolutional network eliminates reliance on rigid boolean logic by dynamically capturing overlapping features within sequences and representing them as weighted nodes in a graph structure ^31^. This process allows a more nuanced and flexible detection of piRNA features that traditional rule-based methods often miss.

Long short-term memory-based models, including LSTM4piRNA, demonstrated high performance in managing large datasets, achieving sensitivity up to 94% and specificity of 91% (Ji B, Luo J, Pan L, Xie X, Peng S. DFL-PiDA: Prediction of piwi-interacting RNA-disease associations based on deep feature learning. In: 2021 IEEE International Conference on Bioinformatics and Biomedicine (BIBM). IEEE; 2021). Their strength lies in modeling sequential dependencies, which is particularly valuable for piRNA detection and classification. Hybrid models that integrate graph convolutional networks and long short-term memory architecture further enhanced predictive accuracy, with studies reporting AUC values as high as 0.96 in detecting piRNA-disease associations related to lung and colorectal cancer ^37^^-^^39^. These hybrid approaches illustrate the power of combining structural and temporal data analysis in piRNA applications.

Despite their complexity, these models hold significant promise for precision oncology. However, simpler models like support vector machines remain relevant, especially when there are limited computational resources. Several studies reported that support vector machines achieved accuracy around 88% in small datasets ^32^^,^^40^^-^^42^. When combined with other models, such as recurrent neural networks or graph convolutional networks, their performance improves while maintaining lower computational costs, making them attractive for implementation in resource-constrained environments ^43^^-^^46^.

This review also highlights gaps in the current body of research. One key issue is the limited reporting on dataset characteristics, including origin, population demographics, or geographical diversity. Most studies did not provide detailed information on the sources of their training datasets, making it difficult to assess generalizability to underrepresented populations, such as those in Latin America. This lack of transparency hinders efforts to adapt artificial intelligence models for global clinical application.

Reproducibility also remains a concern. Many articles failed to share their code or datasets, and some lacked clear documentation of essential methodological components such as hyperparameter optimization, data preprocessing steps, or validation protocols. These omissions reduce the confidence in replicating the reported results and limit their utility for translational research.

While none of the reviewed studies directly addressed ethical or implementation challenges in low-resource healthcare systems, we acknowledge that computationally intensive models, such as long short-term memory or graph convolutional networks, may not be feasible in all contexts. Their integration into public health infrastructure would require financial and technical capacity. The absence of implementation strategies tailored to low- and middle-income countries signals a critical gap. Developing interpretable, cost-effective, and accessible artificial intelligence tools should be a priority.

Lastly, researchers must consider the potential biases introduced during feature selection and preprocessing, particularly in small or unbalanced datasets. Overfitting remains a challenge, and although advanced models often report high accuracy, their success depends on training with diverse, high-quality input data.

In conclusion, this systematic review highlights the transformative potential of artificial intelligence models -particularly machine learning and deep learning ones- in advancing piRNA research. These technologies offer promising avenues for improving detection, diagnosis, and prognostication by extracting complex patterns from high-dimensional biological data. Models such as long short-term memory, graph convolutional networks, and hybrid approaches consistently demonstrate strong predictive performance, while simpler algorithms, like support vector machines, provide practical alternatives in low-resource environments.

However, critical gaps remain. Future research must prioritize methodological transparency, dataset diversity, and equitable implementation strategies. Without addressing these areas, the clinical translation of artificial intelligence-driven piRNA tools will remain limited. By refining model design, sharing reproducible pipelines, and adapting solutions to diverse healthcare systems, artificial intelligence can play a pivotal role in making piRNAbased diagnostics and personalized oncology more accessible, especially in underserved populations.

### Availability of data and materials

The datasets generated or analyzed during the study are available from the corresponding author on reasonable request.

## Supplementary files

**Table:**

Section and Topic	Item #	Checklist item	Location where item is reported
TITLE			
Title	1	Identify the report as a systematic review.	1
ABSTRACT			
Abstract	2	See the PRISMA 2020 for abstracts checklist.	2
INTRODUCTION			
Rationale	3	Describe the rationale for the review in the context of existing knowledge.	3
Objectives	4	Provide an explicit statement of the objective(s) or question(s) the review addresses.	4
METHODS			
Eligibility criteria	5	Specify the inclusion and exclusion criteria for the review and how studies were grouped for the syntheses.	7
Information sources	6	Specify all databases, registers, websites, organizations, reference lists and other sources searched or consulted to identify studies. Specify the date when each source was last searched or consulted.	7
Search strategy	7	Present the full search strategies for all databases, registers and websites, including any filters and limits used.	7
Selection process	8	Specify the methods used to decide whether a study met the inclusion criteria of the review, including how many reviewers screened each record and each report retrieved, whether they worked independently, and if applicable, details of automation tools used in the process.	8
Data collection process	9	Specify the methods used to collect data from reports, including how many reviewers collected data from each report whether they worked independently, any processes for obtaining or confirming data from study investigators, and if applicable, details of automation tools used in the process.	8
Data items	10a	List and define all outcomes for which data were sought. Specify whether all results that were compatible with each outcome domain in each study were sought (*e.g.,* for all measures, time points, analyses), and if not, the methods used to decide which results to collect.	8
	10b	List and define all other variables for which data were sought (*e.g*., participant and intervention characteristics, funding sources). Describe any assumptions made about any missing or unclear information.	8
Study risk of bias assessment	11	Specify the methods used to assess risk of bias in the included studies, including details of the tool(s) used, how many reviewers assessed each study and whether they worked independently, and if applicable, details of automation tools used in the process.	9
Effect measures	12	Specify for each outcome the effect measure(s) (*e.g.,* risk ratio, mean difference) used in the synthesis or presentation of results.	9
Synthesis methods	13a	Describe the processes used to decide which studies were eligible for each synthesis [*e.g*., tabulating the study intervention characteristics and comparing against the planned groups for each synthesis (item #5)].	9
	13b	Describe any methods required to prepare the data for presentation or synthesis, such as handling of missing summary statistics, or data conversions.	9
	13c	Describe any methods used to tabulate or visually display results of individual studies and syntheses.	8
	13d	Describe any methods used to synthesize results and provide a rationale for the choice(s). If meta-analysis was performed, describe the model(s), method(s) to identify the presence and extent of statistical heterogeneity, and software package(s) used.	8
	13e	Describe any methods used to explore possible causes of heterogeneity among study results (*e.g.,* subgroup analysis, meta-regression).	8
	13f	Describe any sensitivity analyses conducted to assess robustness of the synthesized results.	8
Reporting bias assessment	14	Describe any methods used to assess risk of bias due to missing results in a synthesis (arising from reporting biases).	8
Certainty assessment	15	Describe any methods used to assess certainty (or confidence) in the body of evidence for an outcome.	8
RESULTS			
Study selection	16a	Describe the results of the search and selection process, from the number of records identified in the search to the number of studies included in the review, ideally using a flow diagram.	9
	16b	Cite studies that might appear to meet the inclusion criteria, but which were excluded, and explain why they were excluded.	9
Study characteristics	17	Cite each included study and present its characteristics.	9
Risk of bias in studies	18	Present assessments of risk of bias for each included study.	9
Results of individual studies	19	For all outcomes, present, for each study: (a) summary statistics for each group (where appropriate) and (b) an effect estimates and its precision (*e.g.,* confidence/credible interval), ideally using structured tables or plots.	9
Results of syntheses	20a	For each synthesis, briefly summarize the characteristics and risk of bias among contributing studies.	9
	20b	Present results of all statistical syntheses conducted. If meta-analysis was done, present for each the summary estimate and its precision (*e.g*., confidence/credible interval) and measures of statistical heterogeneity. If comparing groups, describe the direction of the effect.	10
	20c	Present results of all investigations of possible causes of heterogeneity among study results.	10
	20d	Present results of all sensitivity analyses conducted to assess the robustness of the synthesized results.	10
Reporting biases	21	Present assessments of risk of bias due to missing results (arising from reporting biases) for each synthesis assessed.	10
Certainty of evidence	22	Present assessments of certainty (or confidence) in the body of evidence for each outcome assessed.	11
DISCUSSION			
Discussion	23a	Provide a general interpretation of the results in the context of other evidence.	13
	23b	Discuss any limitations of the evidence included in the review.	13
	23c	Discuss any limitations of the review processes used.	13
	23d	Discuss implications of the results for practice, policy, and future research.	14
OTHER INFORMATION			
Registration and protocol	24a	Provide registration information for the review, including register name and registration number, or state that the review was not registered.	6
	24b	Indicate where the review protocol can be accessed, or state that a protocol was not prepared.	5
	24c	Describe and explain any amendments to information provided at registration or in the protocol.	3
Support	25	Describe sources of financial or non-financial support for the review, and the role of the funders or sponsors in the review.	9
Competing interests	26	Declare any competing interests of review authors.	5
Availability of data, code and other materials	27	Report which of the following are publicly available and where they can be found: template data collection forms; data extracted from included studies; data used for all analyses; analytic code; any other materials used in the review.	14

From: Page MJ, McKenzie JE, Bossuyt PM, Boutron I, Hoffmann TC, Mulrow CD, *et al*. The PRISMA 2020 statement: An updated guideline for reporting systematic reviews. BMJ. 2021;372:n71. https://doi.org/10.1136/bmj.n71
